# A binuclear vanadium oxyfluoride: di-μ-oxido-bis­[(2,2′-bipyrid­yl)fluorido­oxidovanadium(V)]

**DOI:** 10.1107/S1600536810031302

**Published:** 2010-08-11

**Authors:** Paul DeBurgomaster, Jon Zubieta

**Affiliations:** a Department of Chemistry, Syracuse University, Syracuse, New York 13244, USA

## Abstract

The title compound, [V_2_F_2_O_4_(C_10_H_8_N)_2_], is a centrosymmetric binuclear vanadium(V) species with the metal ions in a distorted octa­hedral environment. The coordination geometries of the symmetry-equivalent V^V^ atoms are defined by *cis*-terminal fluoride and oxide groups, unsymmetrically bridging oxide groups and the N-atom donors of the bipyridyl ligand. The crystal packing is stabilized by weak inter­molecular C—H⋯O and C—H⋯F hydrogen bonds.

## Related literature

For oxyfluorido­molybdates and -­vanadates, see: Adil *et al.* (2010 ▶); Burkholder & Zubieta (2004 ▶); DeBurgomaster & Zubieta (2010 ▶); Jones *et al.* (2010 ▶); Michailovski *et al.* (2006 ▶, 2009 ▶).
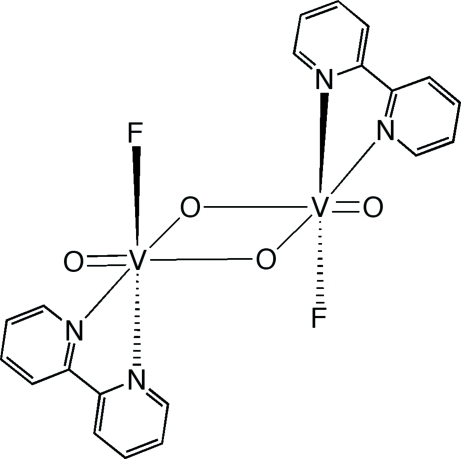


## Experimental

### 

#### Crystal data

[V_2_F_2_O_4_(C_10_H_8_N)_2_]
*M* *_r_* = 516.25
Monoclinic, 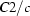

*a* = 9.7526 (6) Å
*b* = 12.6499 (8) Å
*c* = 16.023 (5) Å
β = 92.631 (5)°
*V* = 1974.7 (6) Å^3^
*Z* = 4
Mo *K*α radiation
μ = 1.00 mm^−1^
*T* = 90 K
0.22 × 0.12 × 0.10 mm

#### Data collection

Bruker APEX CCD area-detector diffractometer
Absorption correction: multi-scan (*SADABS*; Bruker, 1998 ▶) *T*_min_ = 0.866, *T*_max_ = 0.905
9692 measured reflections
2406 independent reflections
2357 reflections with *I* > 2σ(*I*)
*R*_int_ = 0.018

#### Refinement

*R*[*F* ^2^ > 2σ(*F* ^2^)] = 0.031
*wR*(*F* ^2^) = 0.079
*S* = 1.13
2406 reflections
145 parameters
H-atom parameters constrained
Δρ_max_ = 0.38 e Å^−3^
Δρ_min_ = −0.38 e Å^−3^

### 

Data collection: *SMART* (Bruker, 1998 ▶); cell refinement: *SAINT* (Bruker, 1998 ▶); data reduction: *SAINT*; program(s) used to solve structure: *SHELXS97* (Sheldrick, 2008 ▶); program(s) used to refine structure: *SHELXL97* (Sheldrick, 2008 ▶); molecular graphics: *CrystalMaker* (Palmer, 2006 ▶); software used to prepare material for publication: *SHELXTL* (Sheldrick, 2008 ▶).

## Supplementary Material

Crystal structure: contains datablocks I, global. DOI: 10.1107/S1600536810031302/om2351sup1.cif

Structure factors: contains datablocks I. DOI: 10.1107/S1600536810031302/om2351Isup2.hkl

Additional supplementary materials:  crystallographic information; 3D view; checkCIF report

## Figures and Tables

**Table 1:** Hydrogen-bond geometry (Å, °)

*D*—H⋯*A*	*D*—H	H⋯*A*	*D*⋯*A*	*D*—H⋯*A*
C1—H1⋯F1^i^	0.95	2.59	3.493 (2)	160
C2—H2⋯O1^ii^	0.95	2.42	3.161 (2)	134
C3—H3⋯F1^iii^	0.95	2.43	3.050 (2)	123
C3—H3⋯F1^ii^	0.95	2.86	3.760 (2)	159
C4—H4⋯O2^iv^	0.95	2.52	3.407 (2)	155
C4—H4⋯F1^iii^	0.95	2.60	3.131 (2)	116
C7—H7⋯O2^iv^	0.95	2.53	3.366 (2)	147

Symmetry codes: (i) 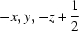; (ii) 
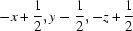
; (iii) 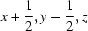; (iv) 
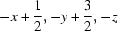
.

**Fig. 1.**
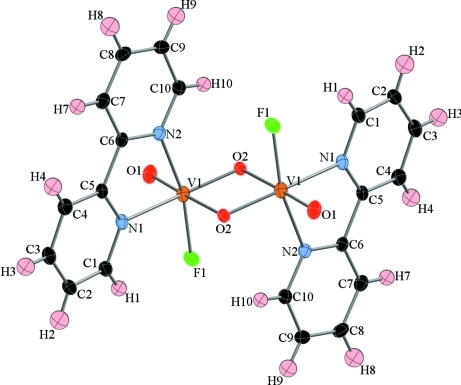
View of the molecular dimer of the title compound, with the atom-labeling scheme and the displacement ellipsoids drawn at the 50% probability level. Color scheme: vanadium, orange; oxygen, red; fluorine, green; nitrogen, light blue; carbon, black; hydrogen, pink.

## supplementary crystallographic information

### Comment

Metal oxyfluorides have attracted considerable contemporary interest as a consequence of their compositional range and structural versatility and for properties such as magnetism, catalysis and non-linear optical behavior (Adil, *et al.* (2010); Burkholder & Zubieta (2004); DeBurgomaster & Zubieta (2010); Jones, *et al.* (2010); Michailovski, *et al.* (2006 and 2009). One approach to the preparation of novel metal oxyfluorides is the exploitation of hydrothermal chemistry where the complexity of the synthetic domain allows incorporation of fluoride into metal oxide frameworks, providing unusual and often unprecedented structures. Furthermore, the metal-oxyfluoride core can be stabilized or modified by the introduction of appropriate coligands, such as organonitrogen donors of the pyridyl family. In the course of our investigations of the hydrothermal chemistry of metal oxides in the presence of fluoride anion, the title compound [V_2_F_2_O_4_(2,2'-bpy)_2_] was isolated.

The compound crystallizes in the monoclinic space group *C*2/*c* with four binuclear molecules per unit cell. The two halves of the binuclear unit are related by a center of symmetry at the mid-point of the V···V vector. The coordination geometry is distorted octahedral with {VFO_3_N} coordination (Fig. 1). The µ-bis-oxo bridging mode produces a V_2_O_2_ rhombus with alternating short-long V—O bond distances of 1.705 (1) Å and 2.293 (1) Å, respectively. The terminal oxo-groups rest in the plane of the V_2_O_2_ rhombus and exhibit a pronounced *trans*-influence on the elongated bridging oxo-group-vanadium distance, V1—O2. The coordination geometry at the vanadium sites also exhibits a fluoride ligand with V—F of 1.806 (1) Å with the V—F vector approximately normal to the V_2_O_2_ rhombus. The V—F vectors of the binuclear unit adopt an *anti*-orientation with respect to the V_2_O_2_ rhombus. The geometry is completed by the nitrogen donors of the 2,2'-bipyridine ligand, which occupy positions *trans* to the short V—O bond of the rhombus and *trans* to the terminal fluoride ligand. The crystal packing is stabilized by weak intermolecular C—H···O and C—H···F hydrogen bonding.

### Experimental

A mixture of V_2_O_5_ (0.062 g, 0.34 mmol), 2,2'-bipyridine (0.320 g, 2.05 mmol), H_2_O (5 ml, 277.5 mmol) and HF (0.200 ml, 5.80 mmol) in the mole ratio 1.00:6.03:1620:17.06 was stirred briefly before heating to 170 °C for 48 h (initial and final pH values of 2.5 and 2.0, respectively). Yellow rods suitable for X-ray diffraction were isolated in 65% yield. Anal. Calcd. for C_20_H_16_F_2_N_4_O_4_V_2_: C, 46.5; H, 3.10; N, 10.8. Found: C, 46.3; H, 3.01; N, 11.0.

### Refinement

All hydrogen atoms were discernable in the difference Fourier map. The hydrogen atoms were placed in calculated positions with C—H = 0.95 Å and included in the riding model approximation with U_iso_(H) = 1.2U_eq_(C).

### Crystal data

**Table:**

[V_2_F_2_O_4_(C_10_H_8_N)_2_]	*F*(000) = 1040.0
*M**_r_* = 516.25	*D*_x_ = 1.737 Mg m^−^^3^*D*_m_ = 1.74 (2) Mg m^−^^3^*D*_m_ measured by flotation
Monoclinic, *C*2/*c*	Mo *K*α radiation, λ = 0.71073 Å
Hall symbol: -C 2yc	Cell parameters from 5752 reflections
*a* = 9.7526 (6) Å	θ = 2.6–28.3°
*b* = 12.6499 (8) Å	µ = 1.00 mm^−^^1^
*c* = 16.023 (5) Å	*T* = 90 K
β = 92.631 (5)°	Rod, yellow
*V* = 1974.7 (6) Å^3^	0.22 × 0.12 × 0.10 mm
*Z* = 4	

### Data collection

**Table:**

Bruker APEX CCD area-detector diffractometer	2406 independent reflections
Radiation source: fine-focus sealed tube	2357 reflections with *I* > 2σ(*I*)
graphite	*R*_int_ = 0.018
Detector resolution: 512 pixels mm^-1^	θ_max_ = 28.1°, θ_min_ = 2.5°
φ and ω scans	*h* = −12→12
Absorption correction: multi-scan (*SADABS*; Bruker, 1998)	*k* = −16→16
*T*_min_ = 0.866, *T*_max_ = 0.905	*l* = −20→21
9692 measured reflections	

### Refinement

**Table:**

Refinement on *F*^2^	Primary atom site location: structure-invariant direct methods
Least-squares matrix: full	Secondary atom site location: difference Fourier map
*R*[*F*^2^ > 2σ(*F*^2^)] = 0.031	Hydrogen site location: inferred from neighbouring sites
*wR*(*F*^2^) = 0.079	H-atom parameters constrained
*S* = 1.13	*w* = 1/[σ^2^(*F*_o_^2^) + (0.0314*P*)^2^ + 4.0741*P*] where *P* = (*F*_o_^2^ + 2*F*_c_^2^)/3
2406 reflections	(Δ/σ)_max_ = 0.001
145 parameters	Δρ_max_ = 0.38 e Å^−^^3^
0 restraints	Δρ_min_ = −0.38 e Å^−^^3^

### Special details

**Table:**

Geometry. All e.s.d.'s (except the e.s.d. in the dihedral angle between two l.s. planes) are estimated using the full covariance matrix. The cell e.s.d.'s are taken into account individually in the estimation of e.s.d.'s in distances, angles and torsion angles; correlations between e.s.d.'s in cell parameters are only used when they are defined by crystal symmetry. An approximate (isotropic) treatment of cell e.s.d.'s is used for estimating e.s.d.'s involving l.s. planes.
Refinement. Refinement of *F*^2^ against ALL reflections. The weighted *R*-factor *wR* and goodness of fit *S* are based on *F*^2^, conventional *R*-factors *R* are based on *F*, with *F* set to zero for negative *F*^2^. The threshold expression of *F*^2^ > σ(*F*^2^) is used only for calculating *R*-factors(gt) *etc*. and is not relevant to the choice of reflections for refinement. *R*-factors based on *F*^2^ are statistically about twice as large as those based on *F*, and *R*- factors based on ALL data will be even larger.

### Fractional atomic coordinates and isotropic or equivalent isotropic displacement parameters (Å^2^)

**Table:**

	*x*	*y*	*z*	*U*_iso_*/*U*_eq_	
V1	0.10805 (3)	1.04160 (2)	0.066763 (17)	0.01424 (10)	
F1	−0.01010 (11)	1.03518 (8)	0.15020 (6)	0.0199 (2)	
O1	0.21267 (13)	1.13285 (10)	0.10042 (8)	0.0205 (3)	
O2	−0.01453 (12)	0.90041 (9)	0.01329 (7)	0.0133 (2)	
N1	0.20945 (14)	0.90735 (11)	0.13175 (9)	0.0144 (3)	
N2	0.25791 (14)	0.97955 (11)	−0.01525 (9)	0.0142 (3)	
C1	0.17578 (18)	0.87555 (14)	0.20780 (11)	0.0176 (3)	
H1	0.1106	0.9156	0.2367	0.021*	
C2	0.23257 (18)	0.78627 (14)	0.24593 (11)	0.0183 (3)	
H2	0.2056	0.7650	0.2996	0.022*	
C3	0.32950 (18)	0.72849 (13)	0.20438 (11)	0.0185 (3)	
H3	0.3696	0.6669	0.2290	0.022*	
C4	0.36682 (18)	0.76235 (14)	0.12620 (11)	0.0180 (3)	
H4	0.4339	0.7248	0.0969	0.022*	
C5	0.30457 (16)	0.85198 (13)	0.09144 (10)	0.0137 (3)	
C6	0.33609 (17)	0.89567 (13)	0.00892 (10)	0.0144 (3)	
C7	0.44046 (17)	0.85774 (14)	−0.03932 (11)	0.0183 (3)	
H7	0.4940	0.7985	−0.0214	0.022*	
C8	0.46484 (17)	0.90815 (15)	−0.11414 (11)	0.0199 (4)	
H8	0.5357	0.8839	−0.1481	0.024*	
C9	0.38475 (18)	0.99419 (15)	−0.13875 (11)	0.0204 (4)	
H9	0.4002	1.0299	−0.1896	0.024*	
C10	0.28181 (18)	1.02718 (14)	−0.08794 (11)	0.0178 (3)	
H10	0.2262	1.0856	−0.1052	0.021*	

### Atomic displacement parameters (Å^2^)

**Table:**

	*U*^11^	*U*^22^	*U*^33^	*U*^12^	*U*^13^	*U*^23^
V1	0.01400 (15)	0.01331 (15)	0.01525 (16)	0.00446 (10)	−0.00118 (10)	−0.00316 (10)
F1	0.0225 (5)	0.0227 (5)	0.0148 (5)	0.0086 (4)	0.0030 (4)	−0.0001 (4)
O1	0.0197 (6)	0.0188 (6)	0.0226 (6)	0.0028 (5)	−0.0028 (5)	−0.0063 (5)
O2	0.0146 (5)	0.0124 (5)	0.0129 (5)	0.0035 (4)	0.0002 (4)	−0.0001 (4)
N1	0.0134 (6)	0.0154 (6)	0.0140 (6)	0.0032 (5)	−0.0016 (5)	−0.0015 (5)
N2	0.0132 (6)	0.0160 (6)	0.0134 (7)	0.0011 (5)	−0.0001 (5)	−0.0018 (5)
C1	0.0180 (8)	0.0197 (8)	0.0150 (8)	0.0044 (6)	0.0000 (6)	−0.0017 (6)
C2	0.0207 (8)	0.0193 (8)	0.0145 (8)	0.0009 (6)	−0.0010 (6)	0.0004 (6)
C3	0.0218 (8)	0.0130 (7)	0.0201 (8)	0.0030 (6)	−0.0037 (7)	0.0003 (6)
C4	0.0183 (8)	0.0151 (8)	0.0207 (8)	0.0049 (6)	0.0006 (6)	−0.0015 (6)
C5	0.0128 (7)	0.0140 (7)	0.0139 (7)	0.0009 (6)	−0.0022 (6)	−0.0028 (6)
C6	0.0140 (7)	0.0140 (7)	0.0150 (8)	0.0002 (6)	−0.0014 (6)	−0.0033 (6)
C7	0.0152 (8)	0.0188 (8)	0.0208 (8)	0.0032 (6)	0.0011 (6)	−0.0033 (6)
C8	0.0151 (8)	0.0257 (9)	0.0192 (8)	0.0012 (7)	0.0040 (6)	−0.0050 (7)
C9	0.0182 (8)	0.0258 (9)	0.0173 (8)	−0.0005 (7)	0.0031 (6)	0.0015 (7)
C10	0.0161 (8)	0.0194 (8)	0.0178 (8)	0.0023 (6)	0.0001 (6)	0.0009 (6)

### Geometric parameters (Å, °)

**Table:**

V1—O1	1.6167 (13)	C2—H2	0.9500
V1—O2^i^	1.7052 (12)	C3—C4	1.388 (3)
V1—F1	1.8064 (11)	C3—H3	0.9500
V1—N2	2.1576 (14)	C4—C5	1.390 (2)
V1—N1	2.2027 (14)	C4—H4	0.9500
V1—O2	2.2934 (12)	C5—C6	1.478 (2)
V1—V1^i^	3.1165 (6)	C6—C7	1.391 (2)
N1—C1	1.338 (2)	C7—C8	1.388 (3)
N1—C5	1.350 (2)	C7—H7	0.9500
N2—C10	1.341 (2)	C8—C9	1.386 (3)
N2—C6	1.353 (2)	C8—H8	0.9500
C1—C2	1.387 (2)	C9—C10	1.386 (2)
C1—H1	0.9500	C9—H9	0.9500
C2—C3	1.389 (2)	C10—H10	0.9500
			
O1—V1—O2^i^	104.55 (6)	N1—C1—H1	118.8
O1—V1—F1	101.51 (6)	C2—C1—H1	118.8
O2^i^—V1—F1	103.81 (5)	C1—C2—C3	118.95 (16)
O1—V1—N2	91.61 (6)	C1—C2—H2	120.5
O2^i^—V1—N2	93.00 (5)	C3—C2—H2	120.5
F1—V1—N2	155.14 (5)	C4—C3—C2	118.86 (16)
O1—V1—N1	97.47 (6)	C4—C3—H3	120.6
O2^i^—V1—N1	154.20 (5)	C2—C3—H3	120.6
F1—V1—N1	84.45 (5)	C3—C4—C5	119.03 (16)
N2—V1—N1	72.87 (5)	C3—C4—H4	120.5
O1—V1—O2	172.25 (6)	C5—C4—H4	120.5
O2^i^—V1—O2	78.60 (5)	N1—C5—C4	121.90 (16)
F1—V1—O2	84.39 (5)	N1—C5—C6	114.17 (14)
N2—V1—O2	81.09 (5)	C4—C5—C6	123.93 (15)
N1—V1—O2	77.95 (5)	N2—C6—C7	121.90 (16)
O1—V1—V1^i^	150.07 (5)	N2—C6—C5	114.31 (14)
O2^i^—V1—V1^i^	46.17 (4)	C7—C6—C5	123.74 (15)
F1—V1—V1^i^	93.36 (4)	C8—C7—C6	118.69 (16)
N2—V1—V1^i^	85.11 (4)	C8—C7—H7	120.7
N1—V1—V1^i^	109.81 (4)	C6—C7—H7	120.7
O2—V1—V1^i^	32.44 (3)	C9—C8—C7	119.33 (16)
V1^i^—O2—V1	101.40 (5)	C9—C8—H8	120.3
C1—N1—C5	118.83 (15)	C7—C8—H8	120.3
C1—N1—V1	122.59 (11)	C10—C9—C8	118.90 (17)
C5—N1—V1	118.50 (11)	C10—C9—H9	120.5
C10—N2—C6	118.87 (15)	C8—C9—H9	120.5
C10—N2—V1	121.00 (11)	N2—C10—C9	122.31 (16)
C6—N2—V1	119.98 (11)	N2—C10—H10	118.8
N1—C1—C2	122.42 (16)	C9—C10—H10	118.8
			
O2^i^—V1—O2—V1^i^	0.0	V1^i^—V1—N2—C6	−112.39 (12)
F1—V1—O2—V1^i^	105.34 (6)	C5—N1—C1—C2	−1.6 (3)
N2—V1—O2—V1^i^	−94.91 (6)	V1—N1—C1—C2	175.09 (13)
N1—V1—O2—V1^i^	−169.16 (6)	N1—C1—C2—C3	1.0 (3)
O1—V1—N1—C1	91.27 (14)	C1—C2—C3—C4	0.3 (3)
O2^i^—V1—N1—C1	−120.14 (16)	C2—C3—C4—C5	−1.0 (3)
F1—V1—N1—C1	−9.65 (13)	C1—N1—C5—C4	0.9 (2)
N2—V1—N1—C1	−179.35 (14)	V1—N1—C5—C4	−175.93 (12)
O2—V1—N1—C1	−95.08 (13)	C1—N1—C5—C6	−178.60 (14)
V1^i^—V1—N1—C1	−101.23 (13)	V1—N1—C5—C6	4.58 (18)
O1—V1—N1—C5	−92.04 (13)	C3—C4—C5—N1	0.4 (3)
O2^i^—V1—N1—C5	56.55 (19)	C3—C4—C5—C6	179.83 (15)
F1—V1—N1—C5	167.04 (12)	C10—N2—C6—C7	0.1 (2)
N2—V1—N1—C5	−2.66 (11)	V1—N2—C6—C7	−175.39 (12)
O2—V1—N1—C5	81.61 (12)	C10—N2—C6—C5	177.71 (14)
V1^i^—V1—N1—C5	75.46 (12)	V1—N2—C6—C5	2.18 (18)
O1—V1—N2—C10	−78.03 (14)	N1—C5—C6—N2	−4.3 (2)
O2^i^—V1—N2—C10	26.64 (13)	C4—C5—C6—N2	176.19 (15)
F1—V1—N2—C10	159.61 (13)	N1—C5—C6—C7	173.19 (15)
N1—V1—N2—C10	−175.34 (14)	C4—C5—C6—C7	−6.3 (3)
O2—V1—N2—C10	104.61 (13)	N2—C6—C7—C8	0.3 (3)
V1^i^—V1—N2—C10	72.17 (13)	C5—C6—C7—C8	−177.03 (15)
O1—V1—N2—C6	97.41 (13)	C6—C7—C8—C9	−0.2 (3)
O2^i^—V1—N2—C6	−157.93 (12)	C7—C8—C9—C10	−0.3 (3)
F1—V1—N2—C6	−25.0 (2)	C6—N2—C10—C9	−0.7 (3)
N1—V1—N2—C6	0.09 (12)	V1—N2—C10—C9	174.79 (13)
O2—V1—N2—C6	−79.96 (12)	C8—C9—C10—N2	0.8 (3)

Symmetry codes: (i) −*x*, −*y*+2, −*z*.

### Hydrogen-bond geometry (Å, °)

**Table:**

*D*—H···*A*	*D*—H	H···*A*	*D*···*A*	*D*—H···*A*
C1—H1···F1^ii^	0.95	2.59	3.493 (2)	160.
C2—H2···O1^iii^	0.95	2.42	3.161 (2)	134.
C3—H3···F1^iv^	0.95	2.43	3.050 (2)	123.
C3—H3···F1^iii^	0.95	2.86	3.760 (2)	159.
C4—H4···O2^v^	0.95	2.52	3.407 (2)	155.
C4—H4···F1^iv^	0.95	2.60	3.131 (2)	116.
C7—H7···O2^v^	0.95	2.53	3.366 (2)	147.

Symmetry codes: (ii) −*x*, *y*, −*z*+1/2; (iii) −*x*+1/2, *y*−1/2, −*z*+1/2; (iv) *x*+1/2, *y*−1/2, *z*; (v) −*x*+1/2, −*y*+3/2, −*z*.

## References

[bb1] Adil, K., Leblanc, M., Maisonneuve, V. & Lightfoot, P. (2010). *Dalton Trans.* pp. 5983–5993. 10.1039/c002333g 20393669

[bb2] Bruker (1998). *SMART*, *SAINT* and *SADABS* Bruker AXS Inc., Madison, Wisconsin, USA.

[bb3] Burkholder, E. & Zubieta, J. (2004). *Inorg. Chim. Acta*, **357**, 279–284.

[bb4] DeBurgomaster, P. & Zubieta, J. (2010). *Acta Cryst.* E**66**, m909. 10.1107/S1600536810026383 PMC3007386 21588148

[bb5] Jones, S., Liu, H., Ouellette, W., Schmidtke, K., O’Connor, C. J. & Zubieta, J. (2010). *Inorg. Chem. Commun.* **13**, 491–494.

[bb6] Michailovski, A., Hussain, F., Springler, B., Wagler, J. & Patzke, G. R. (2009). *Cryst. Growth Des.* **9**, 755–765.

[bb7] Michailovski, A., Rüegger, H., Skeptzakov, D. & Patzke, G. R. (2006). *Inorg. Chem.* **45**, 5641–5652. 10.1021/ic0604359 16813430

[bb8] Palmer, D. (2006). *CrystalMaker* CrystalMaker Software Ltd, Yarnton, England.

[bb9] Sheldrick, G. M. (2008). *Acta Cryst.* A**64**, 112–122. 10.1107/S0108767307043930 18156677

